# FarpScusn: Fully Anonymous Routing Protocol with Self-Healing Capability in Unstable Sensor Networks

**DOI:** 10.3390/s20226683

**Published:** 2020-11-22

**Authors:** Fengyin Li, Ying Wang, Hongwei Ju, Yanli Wang, Zhaojie Wang, Huiyu Zhou

**Affiliations:** 1 School of Computer Science, Qufu Normal University, Rizhao 276826, China; yvonne_wang0806@163.com (Y.W.); darcy_wang@163.com (Y.W.); syncw123@163.com (Z.W.); 2 Experimental Teaching and Equipments Management Center, Qufu Normal University, Rizhao 276826, China; hongweiju@126.com; 3 School of Informatics, University of Leicester, Leicester LE1 7RH, UK; hz143@leicester.ac.uk

**Keywords:** wireless sensor network, anonymous routing protocol, diffie-hellman key exchange algorithm, anonymous identification scheme, self-healing capability

## Abstract

Anonymous technology is an effective way for protecting users’ privacy. Anonymity in sensor networks is to prevent the unauthorized third party from revealing the identities of the communication parties. While, in unstable wireless sensor networks, frequent topology changes often lead to route-failure in anonymous communication. To deal with the problems of anonymous route-failure in unstable sensor networks, in this paper we propose a fully anonymous routing protocol with self-healing capability in unstable sensor networks by constructing a new key agreement scheme and proposing an anonymous identity scheme. The proposed protocol maintains full anonymity of sensor nodes with the self-healing capability of anonymous routes. The results from the performance analysis show that the proposed self-healing anonymity-focused protocol achieves full anonymity of source nodes, destination nodes, and communication association.

## 1. Introduction

With the development of embedded and wireless communication technologies, wireless sensor networks (WSNs) have received increasing attention. Although sensor nodes have limitations of processing volume, battery capacity, and other factors, with the advantages of self-organization, dynamical management, reliability, and data-centric characteristics, they can be rapidly deployed to surveillance areas, such as battlefields, deserts, and so on [1,2]. Therefore, wireless sensor networks will be continuously deployed and developed.

However, because of the large-scale deployment of wireless sensor networks on the Internet, they face critical security and privacy issues. Anonymous technology is an effective method for protecting the privacy of the network users. It hides the relationship between the two parties through certain methods, and protects the identity privacy of the communicators from being hacked [3,4]. During the communication, network users use the anonymous mechanism of network applications to hide their IP addresses and other personal information, and thus protect identity privacy. Anonymity in sensor networks prevents third parties other than the message sender and the base station from knowing the identity of both parties in the communication [5,6]. It includes source nodes anonymity, communication association anonymity, and destination nodes anonymity. In 2004, Roger et al. present Tor, a circuit-based low-latency anonymous communication service [7]. In 2018, Mougy et al. used a cryptographic technology and key distribution algorithms in order to implement the “onion routing” function on the sensor devices, and proposed a privacy protection method for wireless sensor networks based on onion routing [8]. In 2019, Zhou et al. proposed an anonymous routing scheme to protect location privacy in wireless sensor networks by setting a proxy source node and setting a branch area around the base station [9].

Self-healing anonymous routing achieves self-healing on the basis of anonymous routing. In many cases, wireless sensor networks are unstable, and their topologies may change over time. In 2019, Baranov et al. proposed the self-healing concept in routing protocols. We call a routing algorithm “self-healing” if it satisfies the following two conditions:

(1) After the local network topology changes, the route can be modified locally and dynamically, and finally the route is optimized and the total energy consumption is saved.
(2) When the network topology changes, it is not necessary to reinitiate the routing request and route reply, which greatly improves the system efficiency [10].

Self-healing can ensure that routes can effectively adapt to the changes of the network topology on the unstable sensor networks. In 2009, Chen et al. proposed a multi-next hop self-healing routing technology that is based on loop avoidance and local fast self-healing methods e.g., [11]. In 2015, in order to solve the problems of severe routing flooding and poor self-healing performance of existing wireless network routing algorithms, Zhang et al. proposed a centralized self-healing routing algorithm by introducing the idea of centralized routing and multi-route strategy into self-healing routing [12]. In 2019, Baranov et al. proposed a lightweight decentralized adaptive anonymous routing scheme by combining onion routing for the initial global route request and threshold-based secret sharing for the subsequent local route tuning/healing [10].

Most of the existing self-healing anonymous communication protocols in wireless sensor networks cannot provide source node anonymity, destination nodes anonymity, and communication association anonymity at the same time. The attacker can track the source node or the base station according to the identity field in the message. If the identity or location of the source node or the destination node is leaking, then it may cause large damage to the deployed sensor networks [13]. Therefore, it is very important to maintain the anonymity of nodes during data communication [14].

This paper proposes an enhanced anonymous routing protocol with self-healing capability in unstable sensor networks in order to solve the problem of route-failure and weak anonymity in the existing self-healing anonymous routing protocol.The key contributions of this paper are listed, as follows.

(1) A new anonymous identities scheme is proposed. It includes three parts.
  - A novel key distribution and sharing scheme is proposed to provide keys for the following anonymous identity generation and updating process.
  - A new anonymous identity generation scheme is proposed to protect the identity privacy of the sensor nodes.
  - A new anonymous identity updating mechanism is proposed to prevent long-term passive eavesdroppers from inferring network topology through traffic analysis.
(2) Based on the former proposed anonymous identities scheme, a fully anonymous routing protocol with self-healing capability is proposed, which can be applied into unstable sensor networks.

The paper is organized, as follows. In Section 2, we present the fundamental knowledge used in this paper. Section 3 reveals the proposed a self-healing enhanced anonymous routing protocol and its performance analysis. Section 4 concludes the whole paper.

## 2. Preliminary

### 2.1. On-Demand Route Discovery

In on-demand routing protocols, the route from the source node to the destination node is only established when the source node needs to communicate with the destination node [15].

When the network needs to send over data but no available route is found, the source node broadcasts a RREQ (Route REQuest) message to all of its neighbors. After the neighbor node has received the RREQ message, it broadcasts the RREQ message to its neighbors until the destination node is reached. When the RREQ message reaches the destination node, the destination node will answer with a RREP (Route REPly) message. When the source node receives the RREP message, it indicates that the route has been established and it is now ready to communicate.

Once the node receives the RREP message, the routing table is created. A routing table entry is responsible for the routing to reach a destination IP address. Table 1 shows the routing table structure. In order to keep the route up-to-date, the routing table needs to maintain the destination sequence number, so that the expired or damaged route can be quickly recovered.

The maintenance of the neighbor node is mainly implemented by regularly broadcasting the Hello message. When a node is in an active route, it notifies the adjacent node of its own survival by broadcasting a Hello message to the adjacent node. The node first determines whether or not it is in an active route. If so, it turns on the timer, and it checks every HELLO_INTERVAL millisecond whether Hello message or RREQ has been broadcast in the past HELLO_INTERVAL millisecond, and it broadcasts a new Hello message for the other case. If the system has not received a Hello message from the neighbor, then the node considers the neighbor to be invalid and deletes the neighbor from the contacts [16].

When a link fails, all of the adjacent nodes that directly use the link produce a RERR message, and the nodes are then linked to the other nodes. The other nodes will do the same thing until all of the affected nodes receive the RERR (Route ERRor) message. Whether the source node or intermediate node needs to send data again to the affected destination node after the link failure, it needs to send RREQ message again to discover the route to the destination node [17,18].

### 2.2. Homomorphic Encryption

Homomorphic encryption provides a function to process the encrypted data. It can perform mathematical operations without decrypting the data, which is equivalent to performing operations on the original data. An encryption scheme is called a homomorphic encryption scheme if it satisfies the following operations: $E\left(x\right)⊙E\left(y\right)=E\left(x⊗y\right)$,∀x,y∈M, where *E* delegates the encryption algorithm, *M* delegates the space of all the possible messages, and ⊙ and ⊗ delegate two different operations applied to the plaintext field and to the ciphertext field, respectively [19].

If “⊗” delegates an addition operation, the scheme is an addition homomorphism. At the same time, “⊙” means a multiplication operation.

If “⊗” delegates a multiplication operation, the scheme is a multiplication homomorphism. At the same time, ”⊙” means an addition operation.

The Paillier homomorphic encryption scheme is a public key encryption scheme that is based on the security assumption of the decisive combinatorial residue class problem. It is an addition homomorphism [10].

Key generation:

For large prime numbers *p* and *q* with gcd(pq,(p−1)(q−1))=1, calculate n=pq and ω=lcm(p−1,q−1). Then, a random integer $g\in Z_{n^{2}}^{*}$ is selected satisfying $\gcd(n,L\left(pow\left(g,\omega \;\mathrm{mod}\;n^{2}\right)\right))=1$, where the function *L* is defined for each $u\in Z_{n^{2}}^{*}$, as follows: L(u)=(u−1)L(u)=(u−1)nn. Consequently, the public key is $\left(n,g\right)$ and the private key is $\left(p,q\right)$.

Encryption:

The message x is encrypted as follows.

$c=E\left(x\right)=g^{x}r^{n}\left(\;\mathrm{mod}\;n^{2}\right)$, where *r* is a randomly selected number.

Decryption:

Ciphertext $c<n^{2}$ can be decrypted, as below:

Dc=Lcωmodn2Lcωmodn2Lgλmodn2Lgωmodn2modn=x, using the private key $\left(p,q\right)$.

Homomorphic Property:

$E\left(x\right)*E\left(y\right)=\left(g^{x}r_{1}^{n}\left(\;\mathrm{mod}\;n^{2}\right)\right)*\left(g^{y}r_{2}^{n}\left(\;\mathrm{mod}\;n^{2}\right)\right)=g^{x+y}{\left(r_{1}*r_{2}\right)}^{n}\left(\;\mathrm{mod}\;n^{2}\right)=E\left(x+y\right)$, whereas $r_{1}$ and $r_{2}$ are the random numbers chosen to encrypt *x* and *y*, respectively.

Based on public key pair (n,g), over operation “+”, the Paillier encryption scheme can perform special homomorphic operations on plaintexts *x*, *y* and encrypted plaintexts E(x), E(y). The homomorphic operation details are as follows.

(1) $$E\left(x\right)*E\left(y\right)\left(\;\mathrm{mod}\;n^{2}\right)=E\left(x+y\left(\;\mathrm{mod}\;n\right)\right),$$

(2) $$E\left(x\right)*g^{y}\left(\;\mathrm{mod}\;n^{2}\right)=E\left(x+y\left(\;\mathrm{mod}\;n\right)\right),$$

(3) $$E{\left(x\right)}^{y}\left(\;\mathrm{mod}\;n^{2}\right)=E\left(x*y\left(\;\mathrm{mod}\;n\right)\right).$$

We use Paillier cryptosystem to significantly reduce the overall computational load on the network: The relay nodes build a cryptographic onion architecture, which can be decoded at the destination in a single pass of asymmetric decryption while using its private key. Additionally, the lightweight encryption and decryption algorithms are implemented.

### 2.3. Cryptographic System

As an asymmetric cryptographic system, SysGlobal consists of three basic operations: encryption E(), decryption D(), and *m*-bounded homomorphic decomposition, which includes three important functions: Merge(), Sep1(), and Sep2().

Here, we introduce the basic principle of bounded homomorphic decomposition operation. Let m be an integer. An encryption scheme (E,D) is an *m*-bounded homomorphism decomposition scheme, if there exists an *m*-bounded homomorphic decomposition operation composed of Merge(), Sep1() and Sep2() in order to assure the following conclusion hold.

For any x,y∈M s.t. x<m and y<m, taking secret and public key pair (sk,pk) as input, the following equation holds: $Sep1\left(D\left(Merge\left(E\left(x,pk\right),E\left(y,pk\right)\right),sk\right)\right)=D\left(E\left(x,pk\right),sk\right))$ and $Sep2\left(D\left(Merge\left(E\left(x,pk\right),E\left(y,pk\right)\right),sk\right)\right)=D\left(E\left(y,pk\right),sk\right))$.

The Paillier Cryptosystem has a *m*-bounded homomorphic decomposition. In the Paillier Cryptosystem, the functions of Merge(), Sep1() and Sep2() are defined, as follows.

For any m<sqrt(n):

(4) $$Merge\left(x,y\right)=E{\left(x\right)}^{m}*E\left(y\right)\left(\;\mathrm{mod}\;n^{2}\right)=c.$$

(5) $$Sep1\left(D\left(c\right)\right)=D\left(c\right)(div\left.m\right)=x.$$

(6) Sep2(D(c))=D(c)(modm)=y.

where E(x) is the encryption of the message *x* and E(y) is the encryption of the message *y*. *m* is an index and *c* is encrypted ciphertext. When the ciphertext *c* is decrypted, the original messages can be obtained after the division operation of Sep1 and the modulus operation of Sep2.

The proof is conducted, as follows:

Based on the inequations of x<m, y<m, and m<sqrt(n) and the two homomorphic properties of the Pailliar schemeas descripted in Equations (1) and (3) as below,

$E\left(x\right)*E\left(y\right)\left(\;\mathrm{mod}\;n^{2}\right)=E\left(x+y\left(\;\mathrm{mod}\;n\right)\right).$

$E{\left(x\right)}^{y}\left(\;\mathrm{mod}\;n^{2}\right)=E\left(x*y\left(\;\mathrm{mod}\;n\right)\right)$.

We can have:

$D\left(c\right)=D\left(E{\left(x\right)}^{m}*E\left(y\right)\right)=D\left(E\left(x*m\right)*E\left(y\right)\right)=D\left(E\left(x*m+y\right)\right)=x*m+y.$

SysGlobal is used in the RREQ process. The asymmetry in the system SysGlobal is that the public key of the destination node is used in order to encrypt the messages and the private key of the destination node is used to decrypt message.

The other symmetric cryptographic system SysLocal with polynomial time encryption $E_{fast}\left(\right)$ and polynomial time decryption $D_{fast}\left(\right)$ is considered.

SysLocal is used in the RREP process when a reply message is sent from the destination node to the source node. It’s a symmetric encryption algorithm. During the RREQ process, each node stores a key locally and then sends it to the destination node. Subsequently, the destination node can use the received symmetric keys to encrypt the RREP message layer by layer. At last, during the RREP process, each relay node decrypts a layer with a symmetric key stored locally.

## 3. Fully Anonymous Routing Protocol with Self-Healing Capability

In this section, the calculated anonymous identities are used in order to communicate between sensors. It effectively prevents malicious nodes from identifying the sender and the receiver by reading intercepted messages from the network. A fully anonymous routing protocol with self-healing capability is proposed by combining the anonymous identity with the lightweight encryption and decryption algorithms. It achieves full anonymity and self-healing capability in sensor networks.

In sensor networks, the sensor nodes send the collected data to the base stations. The architecture of the on-demand anonymous routing protocol is presented in Section 3.1. The parameters that are required for the system are accounted for in Section 3.2. Generation and sharing of anonymous keys, generation and updating of anonymous identities are introduced in Section 3.3. The establishment of on-demand anonymous routes and the self-healing process of anonymous routes are reported in Section 3.4. The performance analysis results are reported in Section 3.5.

### 3.1. Architecture

In the wireless sensor networks, sensor nodes need to send the data to the base station. In this situation, these sensor nodes act as source nodes and the base stations act as destination nodes. However, in the wireless sensor networks, the source node *S* cannot directly communicate with the destination node *G*, and intermediate nodes are needed in order to form a multi-hop route to relay the information.

This paper presents a routing protocol based on on-demand route discovery. When the source node *S* advocates a transmitting route to the destination node, it sends the routing request message RREQ to all of its neighbors. When an intermediate node receives RREQ, its address is attached to the message, and the information is shared with its neighbors, and so on. When the destination receives the request RREQ, it generates a route reply message RREP. The reply is transmitted to the source node *S* along the reverse route where all of the intermediate nodes have been constructed. The source node *S* thus obtains a multi-hop route to the destination node *G*. Then, the node *S* saves the established route to the routing table. However, as the number of the sessions increases, the routing table is quite large. Node *S* can first use the timeliness of the routing table entry to record only the most recent route. Its implementation can learn from the LRU algorithm (the Least Recently Used Page Replacement Algorithm). The basic idea is to select the record with the longest distance from the current access time and eliminate it. Node *S* set up a record stack, when a record is accessed, immediately push it into the record stack, and check whether or not the same records are just pushed into the top of the stack, if there are, then pull out the original records from the record stack, in order to ensure that the same records are not stacked. When a record is eliminated by the system, a record elimination is always taken from the bottom of the record stack, which is, the eliminated record is the longest unused.

**Figure 1:**
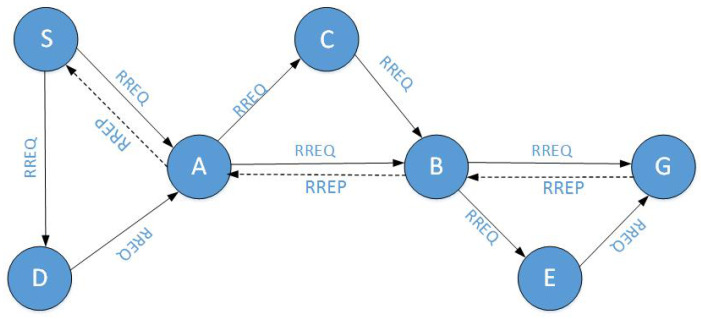
Architecture of On-demand Anonymous Routing Protocol.

In Figure 1, the route S−A−B−G is a multi-hop route to the destination node G established by the on-demand routing strategy.

#### 3.1.1. Anonymity Adversaries

Anonymity means the sender anonymity and the receiver anonymity, used to hide the sender identity and the receiver identity. Only the neighboring nodes in the route ‘know’ each other. By this way, no matter whether it is external or internal and no matter whether it is non-compromised sensors and compromised sensors, any other node cannot identify the source sender and receiver of the message. If a node in the route is compromised by an adversary, it can find its previous hop node, but it cannot find the entire route.

#### 3.1.2. Anonymous Compromise

In the paper, anonymous compromise means that the IDs are revealed, or the anonymous route is revealed. As for the question from the reviewer, if ($x_{1}$,$y_{1}$)($x_{2}$,$y_{2}$)($x_{3}$,$y_{3}$) constitutes a communication route, the adversary can find out a route with nodes in positions ($x_{1}$,$y_{1}$)($x_{2}$,$y_{2}$)($x_{3}$,$y_{3}$), then it is surely a compromised route. The proposed anonymous identities scheme can assure that the real IDs and the anonymous routes are hidden.

### 3.2. System Initialization

Each node is preloaded with the public system parameters ($G_{0}$,$G_{1}$,$G_{2}$,$H_{1}$,$H_{2}$).

In the system initialization phase, we define a public key pre-distribution operation. The public key distribution of all sensor nodes is performed by CA(Certificate Authority). In the proposed routing scheme, CA is acted by the sink node, which is, CA is located in the sink node. All of the nodes communicate with the CA node to obtain all the public keys of other nodes in the pre-distribution operation. The signature of the CA can avoid the man-in-the-middle attack when communicating with the CA authentication center.

The symbols used in the proposed full anonymous communication scheme are defined in Table 2, as follows:

### 3.3. Anonymous Identities Scheme

In this section, a new anonymous identity scheme is proposed through the following three operations. Firstly, each sensor node is assigned a pair of public and private keys for anonymous key exchange and sharing. Secondly, an anonymous identity for each hop is calculated by each sending node. Finally, the anonymous identity is updated by each sending node after the data are forwarded.

#### 3.3.1. Generation and Sharing of Anonymous Keys

The anonymous keys are introduced in order to calculate the anonymous identities of two nodes when they communicate.

Here, we use the DH algorithm to realize the generation and sharing of anonymous $Key_{i,N}$. We introduce a security parameter λ to make the system more secure. The security parameter λ is used to generate the system parameters SP = ($G_{0}$,*p*,*g*) that are needed for the anonymous key generation and sharing. With the increase of security parameters, the length of the key will be increased, and the ability of the system to resist adversary calculation is greatly enhanced.

The system parameter generation algorithm takes as input a security parameter λ. Here, the security parameter delegates the length of the prime number. We have set λ over 384 bit length according to the latest NIST standard (i.e., 512 bit-length). Subsequently, the system parameter generation algorithm generates security parameters. We let G denote a generic, polynomial-time, group-generation algorithm, which takes $1^{\lambda }$ as input and outputs a cyclic group $G_{0}$, with its prime order *p* (||*p*||=λ ), and one of its generators g∈ $G_{0}$.

It chooses the cyclic group ($G_{0}$,*p*,*g*) and returns the system parameters SP = ($G_{0}$,*p*,*g*). In Wireless Sensor Networks, node *N* is a neighbor of Node *i*. First, node *i* and node *N* negotiate with the multiplication group $G_{0}$ of prime order *p*, where the generator of $G_{0}$ is *g*.

Then, node *i* chooses a random natural number $r_{1}$, calculates $X=g^{r_{1}}\;\mathrm{mod}\;p$ and sends *X* to node *N*. Node *N* chooses a random natural number $r_{2}$, calculates $Y=g^{r_{2}}\;\mathrm{mod}\;p$ and sends *Y* to node *i*. Node *i* calculates $Key_{i,N}=Y^{r_{1}}\;\mathrm{mod}\;p=g^{r_{1}r_{2}}\;\mathrm{mod}\;p$ after receiving *Y*, and node *N* calculates $Key_{i,N}=X^{r_{2}}\;\mathrm{mod}\;p=g^{r_{1}r_{2}}\;\mathrm{mod}\;p$ after receiving *X*.

Because of the fact that the DH Algorithm itself does not provide any information about the identity of the two parties, it is vulnerable to the limitations of the man-in-the-middle attack. We introduce an additional auxiliary tool, the CA authentication center in the PKI system, to provide authentication to the public key. Digital Certificate provides a simple way to release public key. All of the participants release their public key by applying to CA for authentication, and verify to CA that they have obtained other party’s public key. Subsequently, both sides of communication can get each other’s public key, and they can verify each other’s identity information by digital signature. Recognizing each other’s identities by verifying the CA signatures, essentially avoiding active man-in-the-middle attack. After the verification is successful, the actual data transfer is carried out.

In this way, the anonymous keys of node *i* and its neighbor node *N* are generated and shared.

#### 3.3.2. Generation of Anonymous Identities

After the anonymous keys between the two nodes are generated and shared, the anonymous identity is generated by calculating the key Hash function of the two nodes. The specific generation process of anonymous identity is as follows.

The anonymous identity of a sensor node is generated by Hash function $H_{1}$.

$H_{1}:{\left\{0,1\right\}}^{*}\to G_{1}$

When nodes *i* and *j* are the neighbors of each other, both nodes *i* and *j* can compute their shared $Key_{i,j}$ using the Diffie–Hellman algorithm proposed in Section 3.3.1. If node *i* expects to communicate with its neighbor node *j*, and it can have an anonymous identity as follows.

(7) $$HIA_{ID,i↔j}=H_{1}\left(Key_{i,j}⊕ID_{i}⊕ID_{j}\right).$$

where ⊕ delegates the XOR operation.

That is, the anonymous identity of node *i* is a hashed result from the shared key between nodes *i* and *j*, the identity of node *i*, and the identity of node *j*.

After the anonymous identity is calculated, both nodes *i* and *j* can share the anonymous identity, which is similar to the “secret signal” agreed by both sides, and realizes the function of identity authentication. Any third party other than these two parties cannot calculate to obtain this “secret signal”.

Nodes *i* and *j* communicate with the shared anonymous identity $HIA_{ID,i↔j}$, which fulfills the identities authentication between two nodes, whilst ensuring that the adversary cannot obtain its real identity.

#### 3.3.3. Update of Anonymous Identities

In order to solve the problem that a long-term passive eavesdropper can infer the real identities of the users by traffic analysis, the sensor nodes update the anonymous identities after the data is forwarded. The details are, as follows.

The anonymous identities of the sensor nodes are updated by hash function $H_{2}$.

$H_{2}:G_{2}\times {\left\{0,1\right\}}^{*}\to {\left\{0,1\right\}}^{n}$.

When nodes *i* and *j* are the neighbors of each other, node *i* computes a pair of shared key $Key_{i↔j}$ with node *j* ij order to update anonymous identity $HIA_{ID,i↔j}$,

(8) $$Key_{i↔j}=H_{2}\left(Key_{i,j}⊕ID_{i}⊕ID_{j}\right).$$

(9) $$HIA_{ID,i↔j}=H_{1}\left(Key_{i↔j}⊕HIA_{ID,i↔j}\right).$$

### 3.4. Fully Anonymous Routing Protocol with Self-Healing Capability

The anonymous identities scheme proposed in Section 3.3 is introduced into this section to implement the fully anonymous routing protocol with self-healing capability.

**Figure 2:**
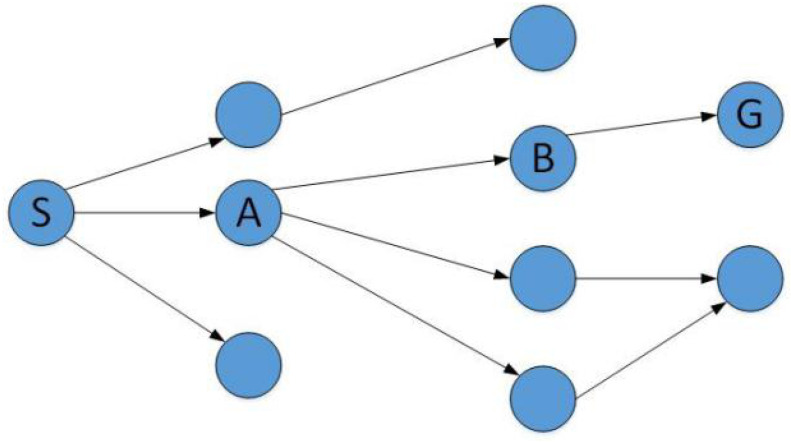
Route Establishment.

When the source node *S* intends to communicate with the destination node *G*, the source node *S* broadcasts the RREQ messages to all of its neighbors, which act as the intermediate nodes in the anonymous route. Each intermediate node broadcasts the RREQ message to its own neighbors, and so on, until the broadcasts the RREQ messages reaches the destination node. Assuming that the route S−A−B−G is the first route to the destination node, and the messages return along this route. The route establishment process is shown in Figure 2.

During the route establishment process, each node creates an anonymous identity to hide its true identity before sending a message to the next node. After the message has been sent, the sending node updates the anonymous identity for the following interaction, in order to avoid the repeated use of the same anonymous identity.

The broadcasting process scheduled by the neighboring nodes is as follows.

When broadcasting the RREQ message, the broadcast node first computes its own sharing anonymous identities. Subsequently, it selects a symmetric key from the SysLocal cryptosystem and encrypts it together its address. At last, it constructs and broadcasts the RREQ message (including session ID, anonymous identity, encrypted symmetric key and address, and the public key of destination node).

When its neighbor node receives the corresponding RREQ message, it first authenticates the validity of the message. If the message is valid, then it will perform the following steps.

Firstly, it stores the corresponding session ID. Secondly, it selects and saves its symmetric key with the destination node locally. Thirdly, it encrypts its own address and symmetric key with the public key of the destination node. Fourthly, it constructs and broadcasts the new RREQ message to its own neighbors.

The following nodes execute the same operations until the RREQ message reaches the destination node.

This protocol is divided into two parts, including the establishment of the on-demand anonymous route and self-healing of the anonymous route. The anonymous identity sets up for each sensor node is used in the RREQ request and RREP reply routes, and finally the route self-healing capability is implemented in the RREP reply route.

#### 3.4.1. Establishment of On-Demand Anonymous Routes

(1) Anonymous RREQ process.

- The source node communicates with its neighbor candidate node
  When source *S* starts to look for a new route for destination node *G*, it will work, as follows.
  Step 1: randomly select a new session ID SessID and a new key $z_{S}$ from the SysLocal cryptosystem, and store it in the local storage in the form of $\left[SessID,z_{S}\right]$.
  Step 2: node *S* creates the one-hop anonymous identity $HIA_{ID,S↔N}=H_{1}\left(Key_{S,N}⊕ID_{S}⊕ID_{N}\right)$ between node *S* and the next neighbor node *N*.
  Step 3: calculate $Key_{S↔N}=H_{2}\left(Key_{S,N}⊕ID_{S}⊕ID_{N}\right)$ and encrypt $pk_{G}$ with $Key_{S↔N}$.
  Step 4: the created anonymous identity is used in order to compose the broadcast RREQ message with the following five attributes:
$E_{pk_{N}}\left(SessID\right)||HIA_{ID,S↔N}\left|\right|E_{Key_{S↔N}}\left(pk_{G}\right)||E\left(S,pk_{G}\right)\left|\right|E\left(z_{S},pk_{G}\right)$
  Where the symbol || means the concatenation operation.
  Let $\alpha =E(S,pk_{G})$, $\mu =E\left(z_{S},pk_{G}\right)$, and source node *S* send the message to the next neighbor node.
  Step 5: update $HIA_{ID,S↔N}$.
- The intermediate node communicates with its neighbor candidate node
  When an intermediate node with address *N* receives a message in the form of $E_{pk_{N}}\left(SessID\right)||HIA_{ID,S↔N}\left|\right|E_{Key_{S↔N}}\left(pk_{G}\right)||\alpha ||\mu$, it will work as follows.
  Step 1: select a new key $z_{N}$ randomly from the SysLocal cryptosystem, and store it in the local storage in the form of $\left[SessID,z_{N}\right]$;
  Step 2: node *N* creates an anonymous ID, the one-hop anonymous identity $HIA_{ID,N↔N_{i}}$ between node *N* and the next neighbor node $N_{i}$.
  Step 3: decrypt $E_{Key_{S↔N}}\left(pk_{G}\right)$ with $Key_{S↔N}$ to retrieve $pk_{G}$, and then use $Key_{N↔N_{i}}$ to encrypt $pk_{G}$.
  Step 4: construct the anonymous identity that communicates with the next neighbor node to form the new RREQ message:
  $E_{pk_{N_{i}}}\left(SessID\right)||HIA_{ID,N↔N_{i}}\left|\right|E_{Key_{N↔N_{i}}}\left(pk_{G}\right)||Merge\left(E\left(N,pk_{G}\right),\alpha \right)\left|\right|Merge\left(E\left(z_{N},pk_{G}\right),\mu \right)$.
  Let $\alpha =Merge\left(E\left(N,pk_{G}\right),\alpha \right)$, $\mu =Merge\left(E\left(z_{N},pk_{G}\right),\mu \right)$, and send the message to the next neighbor node.
  Step 5: update $HIA_{ID,N↔N_{i}}$.
- Destination node receives forwarding data
  When the destination node *G* receives the data α, it uses the theorem $Sep\left(D\left(Merge\left(E\left(x_{1},pk\right),\ldots ,E\left(x_{l},pk\right)\right),sk\right),i\right)=D\left(E\left(x_{i},pk\right),sk\right)$ of bounded decomposition in order to easily recover the whole route $S.N_{1}.N_{2}\cdots$ $N_{m}.G$.
  $$N_{m}:=Sep1\left(D\left(\alpha ,sk_{G}\right)\right);\beta_{1}:=Sep2\left(D\left(\alpha ,sk_{G}\right)\right);$$
  $$N_{m-1}:=Sep1\left(D\left(\beta_{1},sk_{G}\right)\right);\beta_{2}:=Sep2\left(D\left(\beta_{1},sk_{G}\right)\right);$$
  ⋯
  $$N_{1}:=Sep1\left(D\left(\beta_{m-1},sk_{G}\right)\right);\beta_{m}:=Sep2\left(D\left(\beta_{m-1},sk_{G}\right)\right);$$
  $$S:=Sep1\left(D\left(\beta_{m},sk_{G}\right)\right).$$
  Similarly, when the destination node *G* receives the data μ, it can recover the keys $z_{S}.z_{N_{1}}.\cdots .z_{G}$ stored locally at each node of the route. These keys can be used for fast encryption and decryption of any message exchange between the destination node and the corresponding intermediate node (in the context of the current session) during the route reply process.
  In the process of route establishment, lightweight encryption can also be uesd. Paillier homomorphic encryption can carry out mathematical operation on the basis of non-decrypted data, which is equivalent to the operation on the original data [20]. In this case, the system allows for the encrypted data to be processed directly without decryption. Using the Paillier homomorphism property, when $Merge\left(E\left(N,pk_{G}\right),\alpha \right)$ is calculated, it is directly operated on the basis of the encrypted data, and there is no decryption operation for α. On the other hand, when $Merge\left(E\left(z_{N},pk_{G}\right),\mu \right)$ is calculated, it is directly operated on the basis of the encrypted data, and there is no decryption operation for μ.
  Specifically, we encrypt the address of the source node *S* and the address of the next hop *N* of the source node to obtain the ciphertext *c*:
$Merge\left(E\left(N,pk_{G}\right),E\left(S,pkG\right)\right)=E{\left(N,pk_{G}\right)}^{m}*E\left(S,pk_{G}\right)\left(\;\mathrm{mod}\;n^{2}\right)=c.$
  When the destination node receives the ciphertext *c*, the source node and its next hop address can be obtained by decrypting the ciphertext *c* using Sep1 and Sep2.
  The proposed scheme is suitable for wireless sensor networks that are composed of micro devices with very low processing power, storage and battery power.

(2) Anonymous RREP process. 

In the RREP process, a symmetric cryptosystem SysLocal with polynomial time encryption $E_{fast}\left(\right)$ and polynomial time decryption $D_{fast}\left(\right)$ is considered.

When the message arrives at the destination node *G*, *G* needs to send a reply message to the source node. In this process, we achieve full anonymity from the destination node to the source node.

In the route reply process, the destination node uses the key $z_{N_{i}}$ of each node decrypted to encrypt symmetrically. This is done while using the idea of “onion routing”, first in the destination node sequential encrypts *n* layer, and then in the process of the reply layer by layer decryption. The first layer is encrypted with the symmetric key of the source node, and the second layer is encrypted with the symmetric key of the adjacent node of the source node, and so on, until the destination node’s neighbor encrypts the *n*th layer, just like an “onion”.

Destination node *G* sends the data with three attributes to adjacent node $N_{m}$.

$E_{pk_{N_{m}}}\left(SessID\right)||HIA_{ID,G↔N_{m}}\left|\right|E_{fast}\left(K_{N_{m}}\left|\right|N_{m-1}\left|\right|Suffix_{m-1},z_{N_{m}}\right)$

Suffix is defined, as follows:

$Suffix_{i}=E_{fast}\left(K_{N_{i}}\left|\right|N_{i-1}\left|\right|Suffix_{i-1},z_{N_{i}}\right)$, *i* > 1,

$Suffix_{1}=E_{fast}\left(K_{N_{1}}||S||R,z_{N_{1}}\right)$, *R* is a random number.

Where, $K_{N_{i}}$ is a node specific key (or a group of keys) for unlocking additional special routing packets Pkt in the route self-healing process.

After the intermediate node $N_{m}$ receives the data, it can be decrypted with its local symmetric key $z_{N_{m}}$ in order to gain $K_{N_{m}}\left|\right|N_{m-1}\left|\right|Suffix_{m-1}$. The decrypted $K_{N_{m}}$ is stored locally, then $N_{m}$ finds the next node of the reply route via $N_{m-1}$, and sends the decrypted $Suffix_{m-1}=E_{fast}\left(K_{N_{m-1}}\left|\right|N_{m-2}\left|\right|Suffix_{m-2},z_{N_{m-1}}\right)$ combined with SessID and the calculated anonymous identity $HIA_{ID,m↔m-1}$ to the next node $N_{m-1}$, and so on, until the data reaches the source node *S*.

When the data reaches the source node *S*, the source node decrypts it to have a $K_{N_{S}}$, the address of *S* and a random real number *R*, and the process terminates.

#### 3.4.2. Anonymous Route Self-Healing Process

Here, we consider one of the most common topology modifications: in A−B−C route, nodes *A* and *B* are visible to each other, and nodes *B* and *C* are visible to each other. Suppose that we replace the existing route with a new shorter A−C route. However, nodes *A* and *C* know nothing about each other. When nodes *A* and *C* can interact directly, we call this self-healing process [21].

The proposed demand route discovery scheme can find an accessible route when it is needed. Honestly, the self-healing is optimization in the normal environment. While, in the circumstance where some nodes are unreachable, the optimization becomes a self-healing process. The destination node gets the shortcut information through RREQ messages derived from other nodes. If the destination node gets the shortcut before the anonymous route is built, it will certainly construct and send the refreshed RREP message back through the shorter route.

The route self-healing process is implemented in the route reply process, where a special routing packet Pkt is added. This routing package contains some route optimization information shared by the destination node. Initially, a specific packet Pkt is encrypted into $E_{K_{N_{i}}}\left(Pkt\right)$, and the key (the element of $K_{N_{i}}$) used in order to decrypt the special routing packet is distributed by the destination node.

**Figure 3:**
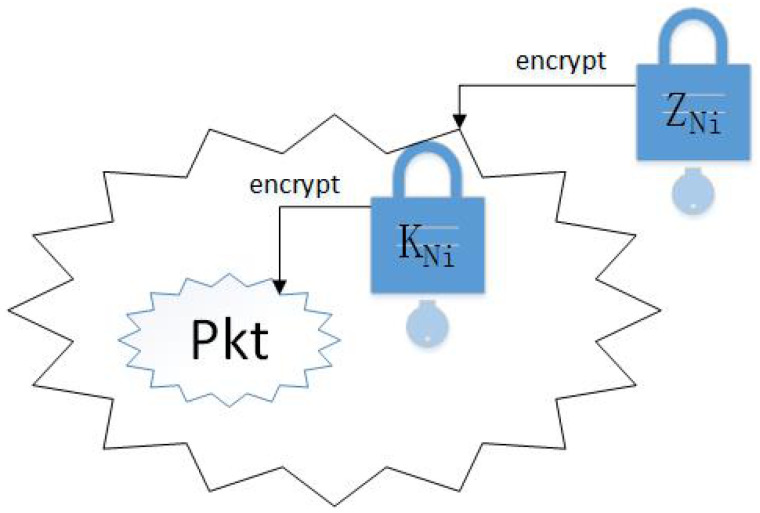
Packet Structure of Encryption Route Information.

In addition, each $E_{K_{N_{i}}}\left(Pkt\right)$ is encrypted by a corresponding symmetric key on the destination node. For example, if the packet is to be used at the node *C*, then the reply message will contain the $E_{fast}\left(E_{K_{N_{i}}}\left(Pkt\right),z_{C}\right)$ data block. Therefore, only the node *C* will be able to decrypt it and get $E_{K_{N_{i}}}\left(Pkt\right)$. The packet structure of encryption route information is shown in Figure 3.

If there is a self-healing route, the information in RREP message will include a special routing packet, which includes the encrypted node IP addresses in the shortcut. That is, when the network topology changes, if there is a shortcut route A−C between node *A* and node *C*, the IP address of the node *A* will be put in the special packet, so that the node *C* can decrypt the IP address of the node *A*.

Specifically, when the destination node finds the shortcut of the route A−C, the destination node puts the information of node *A* (the IP address of the node *A*) in an additional special routing package Pkt, encrypts the routing package into $E_{K_{A}}\left(Pkt\right)$, and only the corresponding $K_{A}$ can decrypt it. The $E_{K_{A}}\left(Pkt\right)$ is encrypted by the symmetric key $z_{C}$ of the corresponding node *C* at the destination node.

When the $E_{fast}\left(E_{K_{A}}\left(Pkt\right),z_{C}\right)$ data block arrives at the node *C* along the route, $z_{C}$ decrypts $E_{K_{A}}\left(Pkt\right)$. When the network topology changes, (1) node *A* enters the wireless detection area of node *C*, (2) node *A* is connected with node *C*, (3) $K_{A}$ can decrypt Pkt, (4) use optimization information in Pkt (the IP address of the node *A*) to optimize, and (5) node *C* can skip *B* to send frame directly to *A*. This completes a route self-healing process.

Now, some intermediate relay nodes have some routing packets with the hidden routing information. Each routing packet is encrypted with $K_{N_{i}}$. To open it, the node must collect the required key. This can only be achieved through direct communication between the nodes.

#### 3.4.3. Specific Implementation Process

For clarity, certain examples present a detailed procedure. According to the previous assumption, route S−A−B−G is the first arrival route, and a concrete implementation process is given. In the process of route establishment, we encrypt data one by one, and finally decrypt it at the destination node. In this section, we implement the full anonymity of the entire route from the source to the destination nodes. The process is as follows:

Assuming the route is established as S→A→B→G.

(1) Anonymous route request message (RREQ) request process 

Step 1: each node enters a session key $z_{N_{i}}$ in the encrypted part of its RREQ message to establish a symmetric encrypted connection with the destination node.

Step 2: each sending node constructs an anonymous identity before each forwarding operation, which prevents the third party other than the sender and receiver from observing the communication between the two nodes.

Step 3: encrypts the node location information at each new relay node, which prevents the receiving candidate node from viewing the route that data forwarding has been circulated from the sender routing.

**Figure 4:**
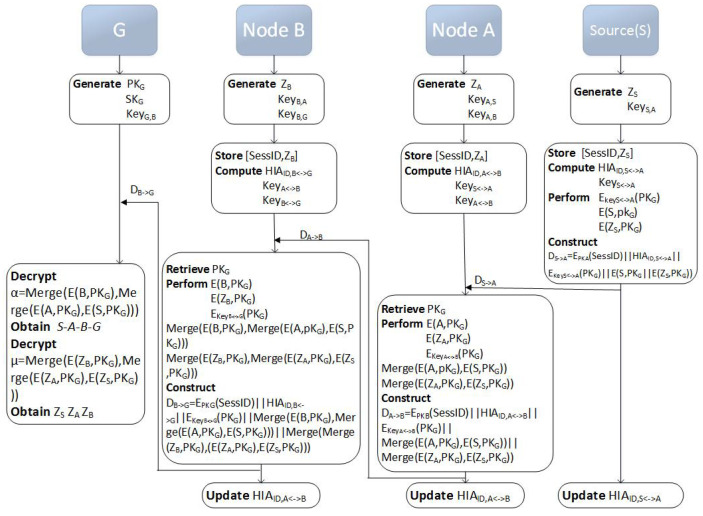
RREQ Process.

Step 4: each sending node updates its own anonymous identity after the data has been sent, which prevents long-term passive eavesdroppers extrapolating the network topology through traffic analysis. The RREQ process is shown in Figure 4.

Conversely, the reply of the base station node (RREP message) is an “onion” and these layers can be “removed” only by using the symmetric password of the corresponding relay node.

(2) Anonymous route reply message (RREP) reply process 

Based on the previous assumption that the route S−A−B−G is the first arrival route, the RREP reply process is G→B→A→S. The details are as follows:

Step 1: node *G* computes $E_{pk_{B}}\left(SessID\right)||HIA_{ID,G↔B}\left|\right|E_{fast}\left(K_{B}\left|\right|A\left|\right|Suffix_{A},z_{B}\right)$, where $Suffix_{A}=E_{fast}\left(K_{A}\left|\right|S\left|\right|Suffix_{S},z_{A}\right)$.

Subsequently, node *G* sends $E_{pk_{B}}\left(SessID\right)||HIA_{ID,G↔B}\left|\right|E_{fast}\left(K_{B}\left|\right|A\left|\right|Suffix_{A},z_{B}\right)$ to node *B*.

At last, node *G* updates $HIA_{ID,G↔B}$.

Step 2: node *B* decrypts $E_{fast}\left(K_{B}\left|\right|A\left|\right|Suffix_{A},z_{B}\right)$ with $z_{B}$, gets $K_{B}$, the address of *A* node and $Suffix_{A}$.

Afterwards, node *B* sends data $E_{pk_{A}}\left(SessID\right)||HIA_{ID,B↔A}\left|\right|E_{fast}\left(K_{A}\left|\right|S\left|\right|Suffix_{S},z_{A}\right)$ to node *A*.

At last, node *B* updates $HIA_{ID,B↔A}$.

Step 3: node *A* decrypts $E_{fast}\left(K_{A}\left|\right|S\left|\right|Suffix_{S},z_{A}\right)$ with $z_{A}$, gets $K_{A}$, the address of the *S* node and $Suffix_{S}$.

Subsequently, node *A* sends data $E_{pk_{S}}\left(SessID\right)||HIA_{ID,A↔S}\left|\right|E_{fast}\left(K_{S}\left|\right|S\left|\right|R,z_{S}\right)$ to *S*.

At last, node *A* update $HIA_{ID,A↔S}$.

**Figure 5:**
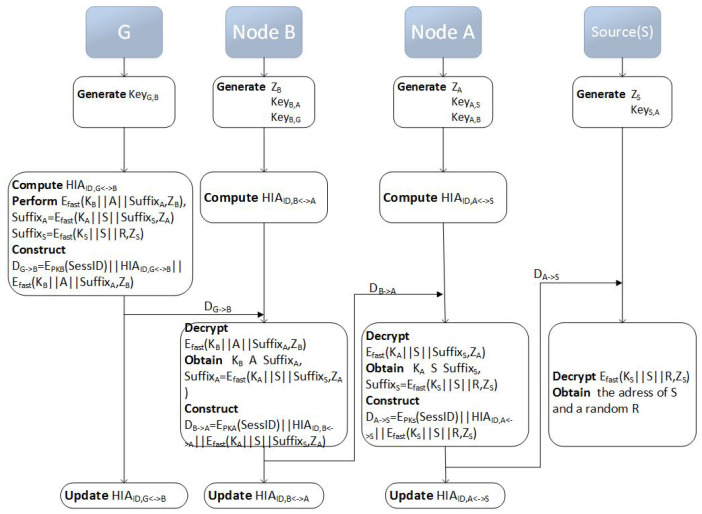
RREP Process.

Step 4: node *S* decrypts $E_{fast}\left(K_{S}\left|\right|S\left|\right|R,z_{S}\right)$ with $z_{S}$, gets its own address and a random number *R*. The RREP process terminates. The RREP process is shown in Figure 5.

(3) Anonymous route self-healing process 

**Figure 6:**
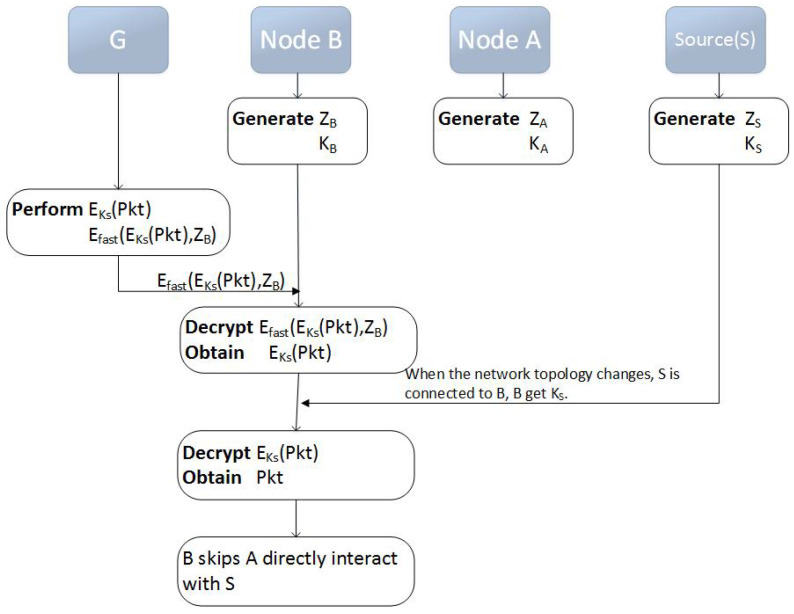
Self-healing Process of Anonymous Routes.

As an example, the route reply process is G→B→A→S, and the destination node has found a shortcut from node *B* to node *S*. The self-healing process is shown in Figure 6.

Step 1: the destination node dispatches a key $K_{N_{i}}$ for each node on the route.

Step 2: ehe destination node places the *S* node’s information in an additional special routing packet called Pkt and encrypts it to $E_{K_{S}}\left(Pkt\right)$, which can only be decrypted by $K_{S}$ (where $K_{S}$ can be the *S* node’s ID).

Step 3: $E_{K_{S}}\left(Pkt\right)$ is encrypted with the symmetric key $z_{B}$ of node *B*, which forms a data block $E_{fast}\left(E_{K_{S}}\left(Pkt\right),z_{B}\right)$.

Step 4: the data block goes one step to *B* along the route, which is G→B→A→S, and it is decrypted by *B* with $z_{B}$ to obtain $E_{K_{S}}\left(Pkt\right)$.

Step 5: if the network topology changes, there is a shortcut from *B* to *S*. Now node *S* is connected to node *B*, and then *B* decrypts $E_{K_{S}}\left(Pkt\right)$ with $K_{S}$.

Step 6: using the optimization information in the Pkt, node *B* skips node *A* and directly interacts with the node *S*.

### 3.5. Performance Analysis

#### 3.5.1. Anonymity

The proposed protocol realizes three kinds of anonymity: source nodes, destination nodes and communication association anonymity. In this section, we will analyze the anonymity performance of the proposed protocol under active attacks. It is assumed that the active attackers can compromise some nodes of a given network and, thus, extract data from these nodes. However, an attacker cannot discover the communication association between the base station, source node, and other nodes in a given network. The detailed analysis is as follows.

(1) Source nodes anonymity 

It is assumed that the active attacker can crack some nodes of a given network and expose data. However, the attacker cannot find the source node. We show the following:

Suppose that the source node *S* passes the underlying data to node *N*,

$$E_{pk_{N}}\left(SessID\right)||HIA_{ID,S↔N}\left|\right|E_{Key_{S↔N}}\left(pk_{G}\right)||E\left(S,pk_{G}\right)\left|\right|E\left(z_{S},pk_{G}\right).$$

This transport hides the source node ID and the address information in $HIA_{ID,S↔N}$ and $E(S,pk_{G})$. Assuming this transmission does not encounter a damaged node before reaching the base station, the attacker cannot find the information of the source node based on the intercepted information.

(2) Communications association anonymity 

When the node *i* communicates with the node *j*, the shared key of nodes *i* and *j* cannot be obtained for the third party node. Therefore, it is not possible to calculate $HIA_{ID,i↔j}=H_{1}\left(Key_{i,j}⊕ID_{i}⊕ID_{j}\right)$, the attack node cannot impersonate the node to communicate, and can not know which two nodes to communicate, which ensures the anonymity of the communication association.

(3) Destination nodes anonymity 

During data transmission, the packet does not contain any information regarding the base station. For passive attackers, it is difficult to find base station nodes from the captured packets. Moreover, the active attacker cannot find the communication association between two nodes as described above, so it is difficult to find the base station, even the active attacker.

In Table 3, we give detailed analysis of anonymity in the proposed protocol and the existing protocols. We infer from Table 3 that APR only implements communication association anonymity, RandomWalk only implements destination nodes anonymity [21,22]. However, our proposed protocol FarpScusn has achieved all three types of anonymity simultaneously.

The improved scheme accomplishes that the adversary cannot determine the identity of the sender and receiver by extracting the messages that are intercepted from the network or by extracting the messages forwarded by its leaking sensor node. The adversary cannot determine whether or not two communication segments (message transmission between two adjacent nodes) belong to the same communication between the source and destination nodes [23].

#### 3.5.2. Self-Healing Capability

Presently, most anonymous routing protocols do not achieve self-healing capability [24]. Traditional anonymous routing methods need to restart new routing requests and route messages recovery when the network topology changes. The proposed protocol implements local self-healing capability by adding a small optimized routing package to the route request message while the network topology changes. Additionally, only minimum necessary information on the network topology is provided to the intermediate nodes.

The self-healing capability analysis can be found in Table 3.

#### 3.5.3. Security

(1) Man-in-the-middle attack 

We introduce the SP = ($G_{0}$,*p*,*g*) that is generated by the system security parameters λ, and the SP is secretly negotiated between the nodes and their neighbors. If the adversary wants to modify or delete the communication information successfully, it must obtain SP.

What is more, we introduce an additional auxiliary tool, the CA authentication center in the PKI system, to provide authentication to the public key. Digital Certificate provides a simple way to release public key. All of the participants release their public keys by applying to CA for authentication, and verify to CA that they have obtained other parties’ public keys. Subsequently, both sides of communication can get each other’s public keys, and they can verify each other’s identity information by digital signatures.

(2) Mutual authentication 

When the node *i* communicates with the node *j*, the anonymous identity $HIA_{ID,i↔j}$ can be calculated by both of the parties. If equal, the certification is successful.

(3) Impersonation attack 

An adversary intercepts some communication contents through channel and pretends to be a legitimate user. First, the adversary does not know which two users are communicating. Second, the adversary cannot calculate the anonymous identity, authenticate, and impersonate.

(4) Forward security and backward security 

The full communication route cannot be obtained by any node, except the destination node.

#### 3.5.4. Simulation

(1) Anonymous Simulation 

In this section, the position of the real sensor node that is detected by the adversary is simulated with Matlab, and the result shows that the proposed protocol can effectively protect the anonymity of the sensor nodes in the communication.

**Figure 7:**
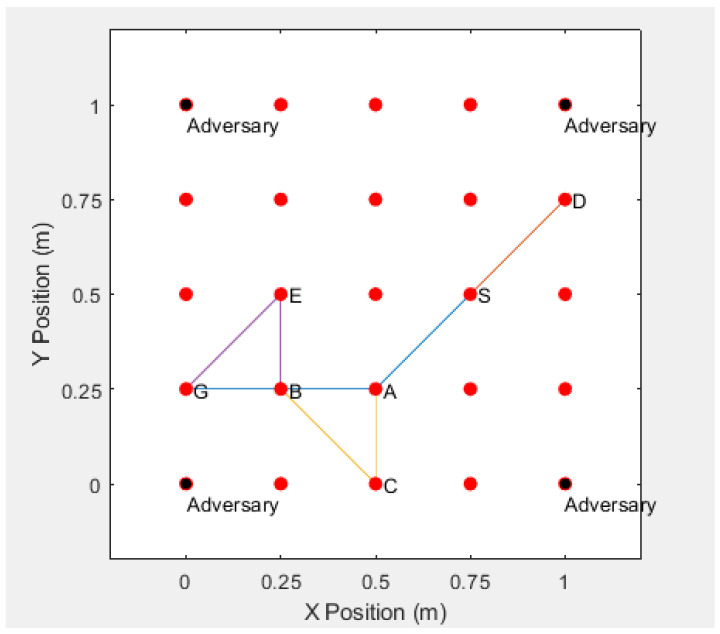
Location of Sensors and Adversaries and a Randomly Generated Network Topology.

In the simulation, we deploy 25 sensor nodes in an area of 1 m × 1 m, where the positions of the four adversaries are deployed at coordinates (0,0),(0,1),(1,0),(1,1), and blind devices (normal sensors that need to guess their positions) are deployed in a square surrounded by the adversaries. We randomly generate a network topology graph from source node *S* to destination node *G* and then generate an anonymous route from *S* to *G*(S−A−B−G), as shown in Figure 7. The red dots are the deployed normal sensor nodes, and the black dots are the adversaries.

**Figure 8:**
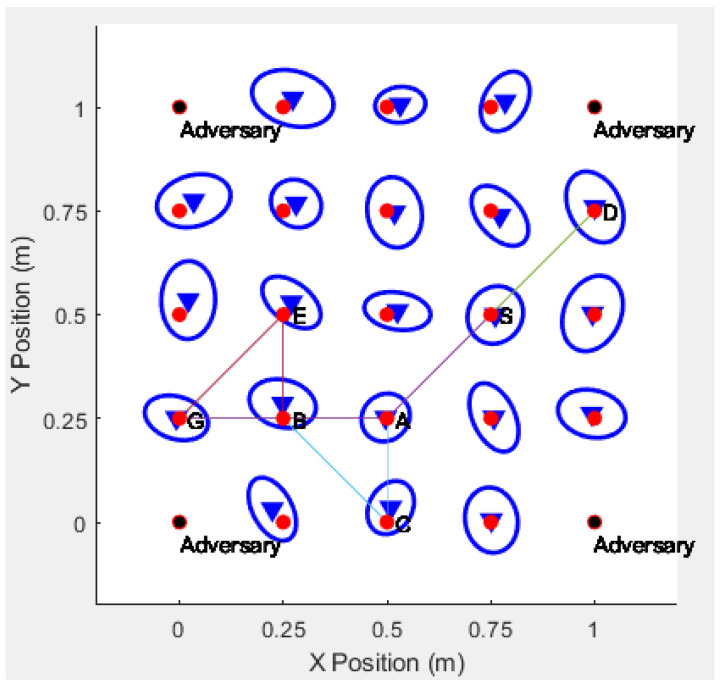
Real Position of Sensor Detected by Adversary.

We consider sensor location estimation when the sensors measure Received Signal Strength (RSS) or Time−Of−Arrival (TOA) between themselves and neighboring sensors. According to the measured RSS or TOA, the adversaries can estimate the positions of sensor nodes by using the Maximum Likelihood Estimation(MLE). The result is that the adversary can accurately guess the actual positions of all the blind devices [25,26], as shown in Figure 8. The blue triangle is the approximate position of the sensor node estimated by the adversary.

**Figure 9:**
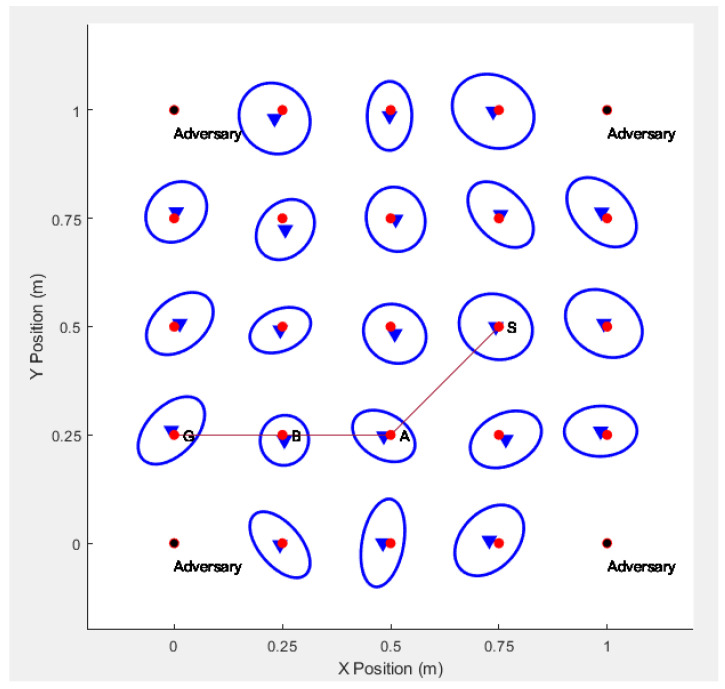
The Route Detected by Adversaries After Intercepting the Sensor’s True Identity.

If real identities are used to communicate between the sensor nodes, the adversaries may obtain the identities of the sensor nodes by intercepting the routing information, and the adversary can accurately decode the route according to the identities of the sensor nodes, and finally find the actual location of the communications sensor. For example, if the adversaries intercept the communication route S−A−B−G, then they can successfully acquire the entire route, which is shown in Figure 9.

**Figure 10:**
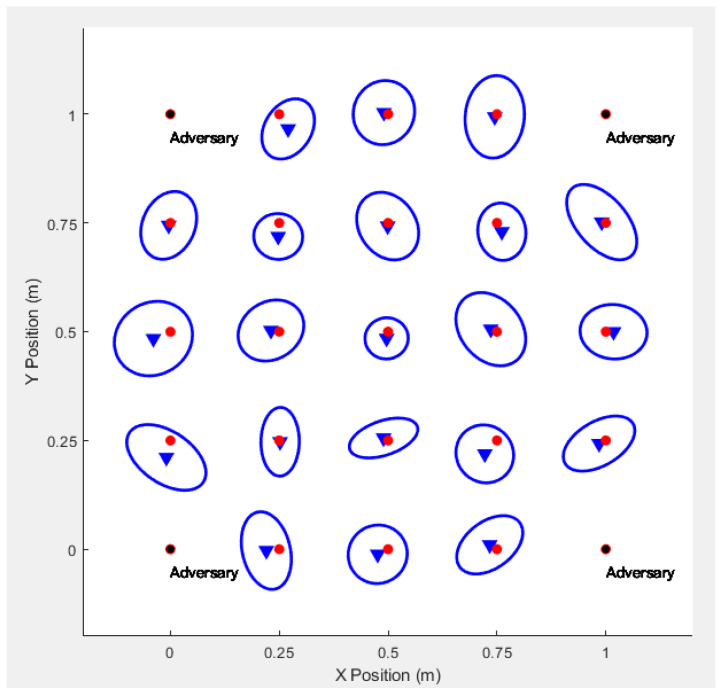
The Adversities Cannot Obtain the Route.

However, the proposed protocol in this paper, while using anonymous identities to communicate, and the adversary cannot intercept the real identities of the sensor nodes, effectively solving the problem, ensures the anonymity of sensor node communication, as shown in Figure 10.

(2) Self-healing simulation 

**Figure 11:**
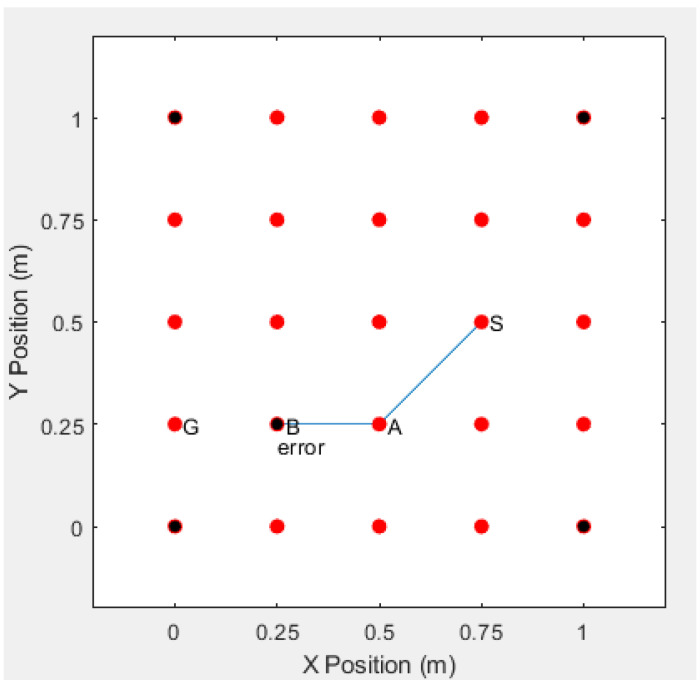
The Error Occurs the Node B.

**Figure 12:**
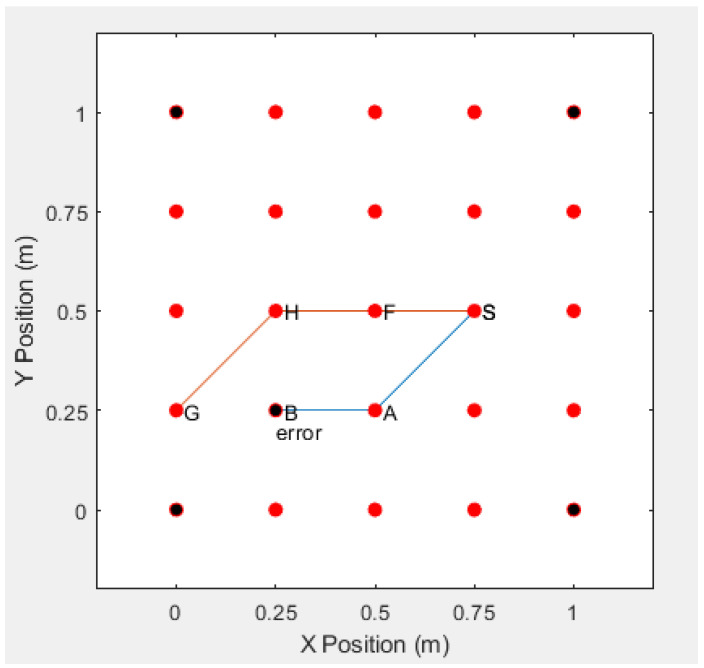
Simulation Without Self-healing Capability.

Under normal circumstances, as shown in the figure below, a node in the S-A-B-G route has an error. If the error occurs at node B, then node S needs to restart the RREQ request in order to establish a new route to reach node G. This leads to complex process for re-connection, as shown in Figure 11 and Figure 12.

On-demand routing can find an accessible route. The destination node determines the updated topology that is based on the received message, and performs route self-healing and optimization. We discuss this in three ways.

**Figure 13:**
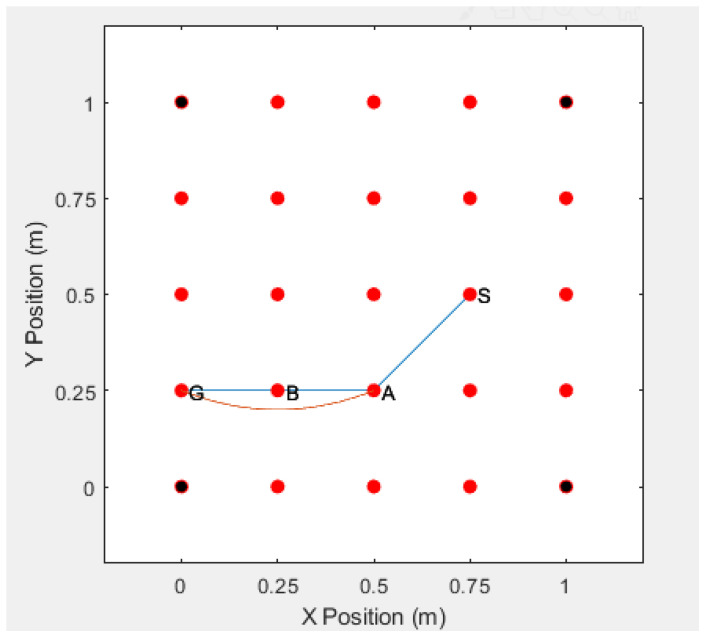
Simulation of Self-healing Capability with Shortcut.

Case 1: according to discussion of Section 3.4.2, when the network topology changes, node A enters the wireless signal area of node *C*. There is an A−C shortcut in the route A−B−C, and node *C* can skip *B* to send the data directly to *A*. When all of the nodes are working properly, if the network topology changes, and a shortcut between *A* and *G* is detected by the destination node, the destination node will immediately carry out the following self-healing process, as shown in Figure 13.

Case 2: if there is a shortcut between node *A* and node *G*, when node *B* goes wrong, the destination node will finds the A−G shortcut, and the self-healing process will be triggered as in the Case 1 and see Figure 13.

**Figure 14:**
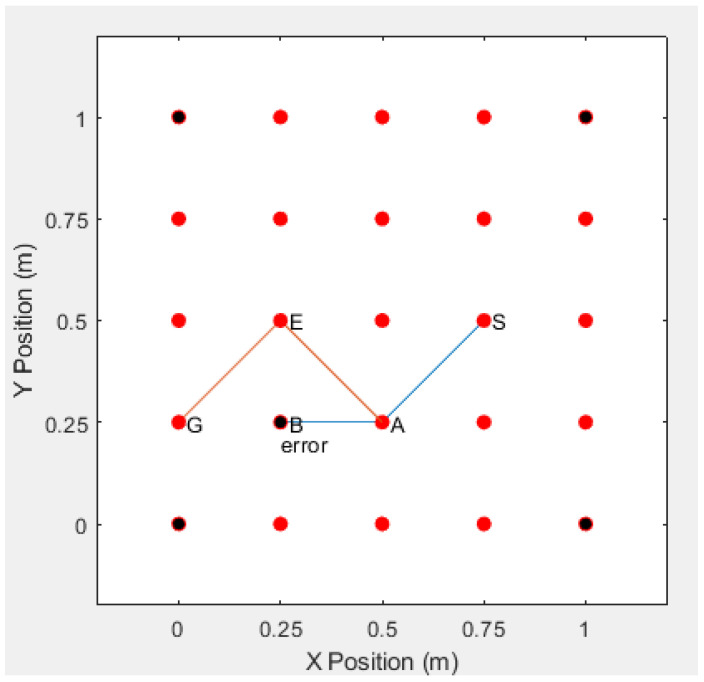
Simulation of Self-healing capability without shortcut.

Case 3: assuming that there is no shortcut between node *A* and node *G*, when node *B* has an error, the destination node will find another accessible route from node *A* to node *G* from other RREQ messages. The new accessible route will use node *E* in (0.25, 0.5) to bypass the error node *B* in the given example, as shown in Figure 14.

## 4. Conclusions

A fully anonymous routing protocol with the self-healing capability in unstable sensor networks has been proposed in this paper. In the proposed protocol, the Paillier cryptosystem was introduced into the secure anonymous communication scheme in order to establish an anonymous identity for every sensor node in the network, which realized full anonymity in unstable wireless environments. Each sensor node communicates with its neighbor nodes using the anonymous identity. Each node encrypts its address information with the public key of the destination node, and sends it to the next node in the anonymous route. After the routing packet has arrived at the destination node, the destination node decrypts the entire route with its corresponding private key. Security analysis shows that the proposed protocol realizes source node, destination node, and communication association anonymity. This proposed protocol is suitable for protecting the anonymity of sensor nodes and it can prevent the adversary from finding the actual nodes such as source node or base station by analyzing the network traffic. The protocol uses a special lightweight implementation tool for wireless sensor networks with low processing power, storage space, and battery power. In the future, we will continue to study on lower energy consumption and safer routing strategies.

## Figures and Tables

**Table 1:** Routing Table Structure.

The Serial Number Of The Destination	The Destination IP Address	The Number of Hops Required to Reach the Destination
The symbol of the legitimate destination serial number		
Other status and routing symbol bits		
Network Interface		
Next hop		
Survival time		

**Table 2:** Symbols definition.

Symbol	Meaning
$Key_{i,N}$	Paired keys shared by node i and neighbor node *N*
$HIA_{ID,i↔j}$	One-hop anonymous identity shared between node *i* and node *j*
$Key_{i↔j}$	The shared key between node *i* and node *j* used to update their anonymous identities
$D_{i\to j}$	Data transferred from node *i* to node *j*
$H_{1}$,$H_{2}$	Hash functions
$pk_{G}$	Public key of destination node *G*
$sk_{G}$	Private key of destination node *G*
*R*	A random number
*S*	The source node with address *S*
*G*	The destination node with address *G*
$z_{N_{i}}$	A symmetric key of $N_{i}$ used in RREP process
SessID	A unique Session ID used in RREQ and RREP processes
$K_{N_{i}}$	The key of $N_{i}$ used to unlock additional routing packets
$ID_{i}$	The real ID of node *i*
λ	security parameter
$G_{0}$,$G_{1}$,$G_{2}$	cyclic group
*p*	prime order
*g*	generator

**Table 3:** Performance Analysis.

Protocol	Source Nodes Anonymity	Communications Association Anonymity	Destination Nodes Anonymity	Self-Healing
FarpScusn	✓	✓	✓	✓
APR	×	✓	×	×
RandomWalk	×	×	✓	×
